# The role of growth factors on hepatic damage in rats with obstructive jaundice

**DOI:** 10.1186/s40064-016-2919-5

**Published:** 2016-08-05

**Authors:** Ozgur Turk, Bartu Badak, Ersin Ates, Emine Dundar, Emine Sutken

**Affiliations:** 1 Department of General Surgery, Private Esentepe Hospital, Bursa, Turkey; 2 Department of General Surgery, Eskisehir Osmangazi University Medical Faculty, Eskisehir, Turkey; 3 Department of Pathology, Eskisehir Osmangazi University Medical Faculty, Eskisehir, Turkey; 4 Department of Biochemistry, Eskisehir Osmangazi University Medical Faculty, Eskisehir, Turkey

**Keywords:** Obstructive jaundice, Hepatic injury, Growth factors

## Abstract

In this study, we investigated the affect and the role of growth factors on liver damage. 110 Sprague–Dawley rats were divided into 11 groups: a sham group, a control group, HGF, EGF, IGF, TGF groups of irreversible jaundiced rats and a control group and HGF, EGF, IGF, and TGF groups of reversible jaundiced rats (n = 10). In the irreversible jaundiced groups, the common bile duct was explorated, double ligated, and cut. 150 μg/kg/day HGF, 5 μg/kg/day EGF, 5 μg/kg/day IGF, and 5 μg/kg/day TGF β-1 were injected intraperitoneally after the seventh post-operative day. In the reversible jaundiced group, the common bile duct was ligated and the ligation was resolved on the seventh post-operative day. For 5 days, growth factors were injected at the same dose. Ductal proliferation scores significantly decreased after growth factor administration in the EGF-A and TGF-A groups. Furthermore, ductal proliferation was decreased in the TGF-B group. As a result of this study, HGF was effective in the irreversible jaundiced groups and ineffective in the reversible jaundice groups. EGF was effective in the reversible jaundiced groups and ineffective in the irreversible jaundiced groups. In both the irreversible jaundiced and reversible jaundiced groups, IGF was ineffective, although TGF β-1 was effective. We believe that these results arise from the positive effects of effective doses of growth factor on liver damage.

## Background

Common bile duct stones, cholangiocellular carcinoma, pancreatic cancer, inflammation, trauma and other mechanical obstructions of the bile duct could result by obstructive jaundice. Due to obstruction of the bile duct jaundice occurs and follows by cholestatic liver damage. Furthermore, as a result of cholestatic liver damage, stellate cells activate and hepatocellular necrosis and bile duct epithelial proliferation occurs. As a result of jaundice inflammation occurs in the liver tissue and due to the release of the inflammatory cytokines and free oxygen radicals liver injury starts. If the jaundice keeps on during 48 h and more, early epithelial proliferation of biliary tract starts. Progression of epithelial proliferation continues during 5 days and makes a peak; after 5 days epithelial proliferation continues at a basal rate. Effects of obstructive jaundice on liver tissue can occur during the first 7 days that can be called the acute period of cholestatic liver injury. The period of liver injury occurred longer than 7 days called as chronic cholestatic period (Georgiev et al. 2008). Cytotoxic biliary acids are responsible from the liver injury and fibrosis (Parola et al. 1996). Jaundice could be presented in different clinical manifestation such as renal failure, coagulation disorders, malnutrition, sepsis, and an inability to heal wounds (Long et al. 2015; Li et al. 2015). Patients who admitted to the hospital because of obstructive jaundice are commonly treated by percutaneous external biliary drainage or internal endoscopic biliary drainage as soon as after the diagnosis of severe obstructive jaundice. Biliary drainage is more effective then medical treatment. Endoscopic biliary drainage is more effective than percutaneous external drainage because of high rate of complications. Bleeding, hemobilia, bile leakage, biliary fistula and peritonitis may be listed among the complications of percutaneous biliary drainage (Pan et al. 2014).

Before surgical treatment surgeons must pay attention to protect patient from liver injury and during preoperative period jaundice must be resolved. Posticteric injury occurs in liver tissue during the chronic cholestatic period after the drainage of bile (Chen et al. 2015). Experimental CBD (common bile duct) ligation in rats is a reliable model represents the cholestatic liver disease in humans. By using the modifications of this experimental model, a newly reversible bile duct ligation model has been developed to investigate changes in posticteric liver damage (Oruc et al. 2009). However; additional studies are required to determine the changes in liver tissue during the posticteric period and the factors affecting healing of cholestatic liver injury, and hepatocyte regeneration. In recent studies, positive effects of growth factors have been detected in liver regeneration. In particular, a mitogenic effect of hepatocyte growth factor (HGF) plays a protective role in necrotic and apoptotic impacts of bile acids on cholestatic livers. Although the expression of HGF results in increased CBDs in ligated rats; therapeutic effects of HGF are still being investigated (Xia et al. 2006). High serum HGF levels were reported in patients admitted to the hospital because of jaundice. Following external biliary drainage, the serum HGF level decreased (Higaki et al. 1999). Epidermal Growth Factor (EGF) has protective effects against carbon tetrachloride-induced hepatic injury (Berlanga et al. 1998). Circulating and locally acting isoforms of insulin-like growth factors protect cholangiocytes from cholestatic injury (Gatto et al. 2008). The protective and therapeutic effects of growth factors on cholestatic liver injuries during the development and release of jaundice merits further investigations (Kedarisetty et al. 2014). The aim of this study is to determine the role of growth factors in protecting the development of cholestatic liver injury and the positive effects on liver regeneration. In addition, we tried to define the effectiveness of growth factors on posticteric injury and anti-inflammatory events during the resolution of jaundice. It is sure that primary method to solve jaundice is biliary drainage and growth factor may contribute to prevent hepatic damage.

## Methods

### Animals

All of the experimental studies were conducted in the laboratory of Eskisehir Osmangazi University’s Medical and Surgical Research Center with the approval of the Ethics and Research Committee of Eskisehir Osmangazi University. In this study, 110 Sprague–Dawley female and male rats weighing 230–260 g were used. The rats were kept at room temperature and in a light–dark cycle in individual plastic cages; there was free access to water and food for 7 days before the experimental procedure.

### Experimental design and surgical procedure

The animals were divided into 11 groups, with at least 10 rats in each group. After eight hours, the rats were administrated general anesthesia using 50 mg/kg sodium pentothal (Pental Sodium, İ.E, Ulagay, Turkey). The rats underwent a midline laparotomy incision by using an aseptic technique. In the sham group, the CBD was isolated from the surrounding tissues by suspending the duodenum (without any additional procedures); the abdominal incision was closed using 3/0 silk sutures. Liver tissues and blood samples were taken on the 7th day of the operation by a relaparotomy. An irreversible CBD ligation model was performed in the HGF A, EGF A, Insulin-like Growth Factor (IGF) A, Transforming Growth Factor (TGF) A, and Control A groups. In the irreversible jaundice groups, the CBD proximal and distal segments were double ligated using 4/0 silk sutures. The CBD cut between the ligations. In the irreversible groups (HGF A, EGF A, IGF A, and TGF A), the rats underwent a growth factor treatment 2 days after the surgical procedure during 5 days via intraperitoneal injection. We used 150 µgr/kg/day HGF (Catalog Number H1404, Sigma-Aldrich, Inc., Turkey), 150 µgr/kg/day EGF (Catalog Number E9644, Sigma-Aldrich, Inc. Turkey), 150 µgr/kg/day IGF (Product Number I3769, Sigma-Aldrich, Inc. Turkey), and 150 µgr/kg/day TGF ß (TGF-b1 human, Catalog Number T7039, Sigma-Aldrich, Inc. Turkey). After treatment, liver tissue and blood samples was taken by relaparotomy.

A reversible CBD ligation model was used in the HGF B, EGF B, IGF B, TGF B, and Control B groups. The CBD was dissected gently from the surrounding tissues and a 1–2 cm segment was released and prepared for ligation. A silicone tube clung around the prepared segment and a silicone band was double wrapped around the tube. The silicone band was tightened enough to stop the flow of bile right into the duodenum and a metal mini clip was placed below the silicone band. The goal of the procedure was to perform a procedure that would reliably and simply reverse jaundice without wounding the CBD and duodenum while releasing the ligation. On the 7th day of the operation, the CBD dissected, the adhesions divided into and the clip at the root of the band was gently removed. The silicone band was also taken out (Kirkland et al. 2010). All treatment groups in the reversible jaundice groups (HGF B, EGF B, IGF B, and TGF B) underwent growth factor treatments with the same dose as the irreversible groups for 5 days. At the end of the treatment liver, tissue and blood samples were taken by relaparotomy.

### Biochemical analysis

Measurement of malondialdehyde (MDA) was made using the thiobarbituric acid method (Ohkawa et al. 1979). The total antioxidant capacity measured using a commercial Antioxidant Assay kit (Antioxidant Assay Kit, Catalog Number CS0790, Sigma-Aldrich, Inc.). Liver tissue samples were homogenized on cold ice and buffered and then centrifuged at 12,000*g* for 15 min at 4 °C. The supernatant was removed for study and placed on plates. The principle of the antioxidant assay relies on the formation of a ferryl myoglobin radical from metmyoglobin and hydrogen peroxide. A soluble chromogen, green in color, can be determined spectrophotometrically at 405 nm (Huang et al. 2005). The blood samples were centrifuged at 5000*g* at 4 °C and the supernatants were removed. Serum Alanine aminotransferase (ALT), Aspartate Aminotransferase (AST), Alkaline phosphatase (ALP), Gamma-glutamyl transpeptidase (GGT), and total bilirubin and direct bilirubin levels were determined.

### Pathological staining for liver injury

All tissues were fixed in 10 % neutral buffered formalin. Five-micron sections from the paraffin-embedded tissue were stained with hematoxylin and eosin and Masson’s Trichrome. Histopathological assessment was accomplished with a quantitative method. We used a scoring system for the ductal proliferation under 10× and 40× light microscopy (Olympus Bx40, Japan) (Sheen-Chen et al. 2006; Miyoshi et al. 1999).

### Statical analysis

For the biochemical results, a one-way analysis of variance (one-way ANOVA) was applied. In this test, for multiple comparisons, the Turkey HDS method was used. For the histopathological scoring results, Mann–Whitney U test was used. Turkey HDS method and Kruskal–Wallis test were used to determine differences between the groups.

## Results

### Biochemical analysis

In both control groups, serum biochemical parameters were used to determine the liver injury. The biochemical parameters of the reversible and irreversible groups were compared and the reversible groups showed significantly larger parameters (p < 0.05). ALP levels were higher in the HGF A group (p < 0.05). AST levels were higher in the TGF B group (p < 0.05). In the EGF B group, the total serum bilirubin level was higher than in the EGF A group and the direct bilirubin level was significantly lower in the EGF A group and higher in the HGF A and IGF A groups. In the irreversible jaundiced group, the total serum bilirubin levels were similar, excluding the EGF B group (p < 0.05). Furthermore, there was no difference between all groups in GGT levels. Biochemical results were listed in Table 1.

**Table 1:** Biochemical results of the all groups

	ALT	AST	ALP	Total bilirubin	Direct bilirubin	GGT
Sham	49.70 ± 6.00	149.80 ± 18.93	258.40 ± 23.82	0.09 ± 0.02	0.09 ± 0.15	7.70 ± 1.89
HGF A	148.60 ± 74.97	618.60 ± 376.55	880.30 ± 407.10	10.77 ± 2.19	8.80 ± 2.02	32.40 ± 6.70
EGF A	145.50 ± 65.36	697.60 ± 298.97	664.10 ± 237.57	9.07 ± 2.12	5.58 ± 2.57	39.10 ± 10.18
IGF A	220.90 ± 136.43	820.90 ± 621.50	856.00 ± 493.76	11.40 ± 1.74	8.63 ± 2.12	32.10 ± 4.93
TGF A	148.50 ± 61.91	478.90 ± 243.28	815.70 ± 267.67	11.53 ± 1.82	7.75 ± 1.98	31.60 ± 7.63
Control A	211.30 ± 80.81	622.80 ± 187.04	758.50 ± 192.11	10.90 ± 1.37	8.42 ± 1.25	32.10 ± 5.51
HGF B	731.50 ± 399.44	681.70 ± 226.30	505.90 ± 302.56	12.14 ± 1.70	9.17 ± 1.84	26.60 ± 5.04
EGF B	597.10 ± 361.76	711.50 ± 237.16	633.90 ± 247.38	12.46 ± 1.79	9.74 ± 1.54	38.10 ± 5.20
IGF B	568.70 ± 219.44	730.50 ± 185.72	677.90 ± 290.03	12.04 ± 0.97	9.21 ± 1.09	29.20 ± 4.37
TGF B	682.00 ± 199.50	973.30 ± 571.17	550.80 ± 316.82	10.79 ± 3.03	8.07 ± 2.86	31.70 ± 3.23
Control B	387.70 ± 294.53	826.60 ± 410.62	779.10 ± 283.79	11.02 ± 1.42	8.30 ± 1.39	31.10 ± 5.90

### Liver tissue antioxidant and MDA assay

**Fig. 1:**
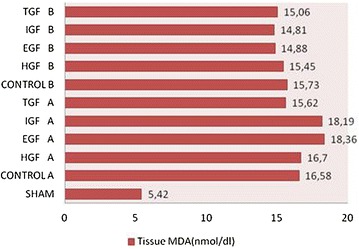
Tissue MDA levels in all groups. EGF A groups MDA level was significantly high than EGF B group. IGF A group’s MDA level was significantly high than IGF B group. (p < 0.05)

**Fig. 2:**
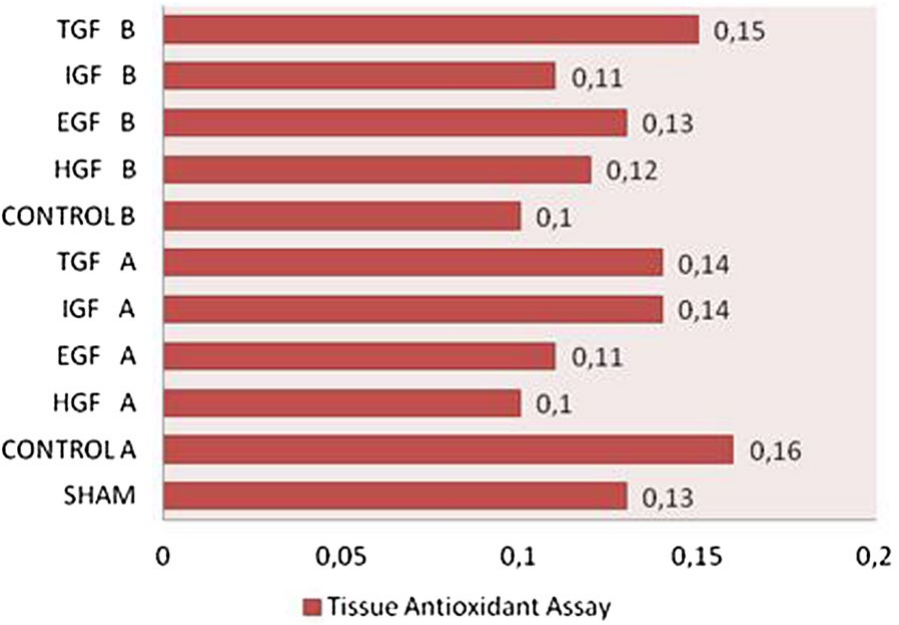
Liver tissue antioxidant assay levels. In all groups there was no significantly difference in antioxidant assay levels

The liver tissue MDA level was significantly higher in the irreversible jaundiced EGF A group than in the EGF B group. In the IGF B group, the MDA level was lower than in the IGF A group (p < 0.05). In the HGF and TGF treatment groups, MDA levels were similar for both the reversible and irreversible jaundiced groups (Fig. 1). Also, there was no significant difference among all groups in terms of liver tissue antioxidant assay levels (Fig. 2). Antioxidant capacity and MDA levels of the liver tissue were listed in Table 2.

**Table 2:** Pathological examination score, tissue oxygenase level and MDA level of the all groups

Groups	Pathology score	Tissue oxygenase level	MDA level
Sham	1 ± 0.5	0.13 ± 0.06	5.42 ± 1.25
HGF A	4.50 ± 0.71	0.10 ± 0.04	16.70 ± 1.61
EGF A	3.90 ± 0.32	0.11 ± 0.03	18.36 ± 1.53
IGF A	5.00 ± 1.05	0.14 ± 0.07	18.19 ± 1.76
TGF A	3.80 ± 1.14	0.14 ± 0.03	15.62 ± 1.86
Control A	5.30 ± 0.82	0.16 ± 0.07	16.58 ± 1.07
HGF B	4.40 ± 0.52	0.12 ± 0.04	15.45 ± 1.64
EGF B	4.00 ± 0.94	0.13 ± 0.06	14.88 ± 2.26
IGF B	5.20 ± 0.63	0.11 ± 0.03	14.81 ± 3.25
TGF B	3.30 ± 0.82	0.15 ± 0.04	15.06 ± 2.09
Control B	4.60 ± 0.70	0.10 ± 0.03	15.73 ± 1.89

### Histopathological evaluation

**Fig. 3:**
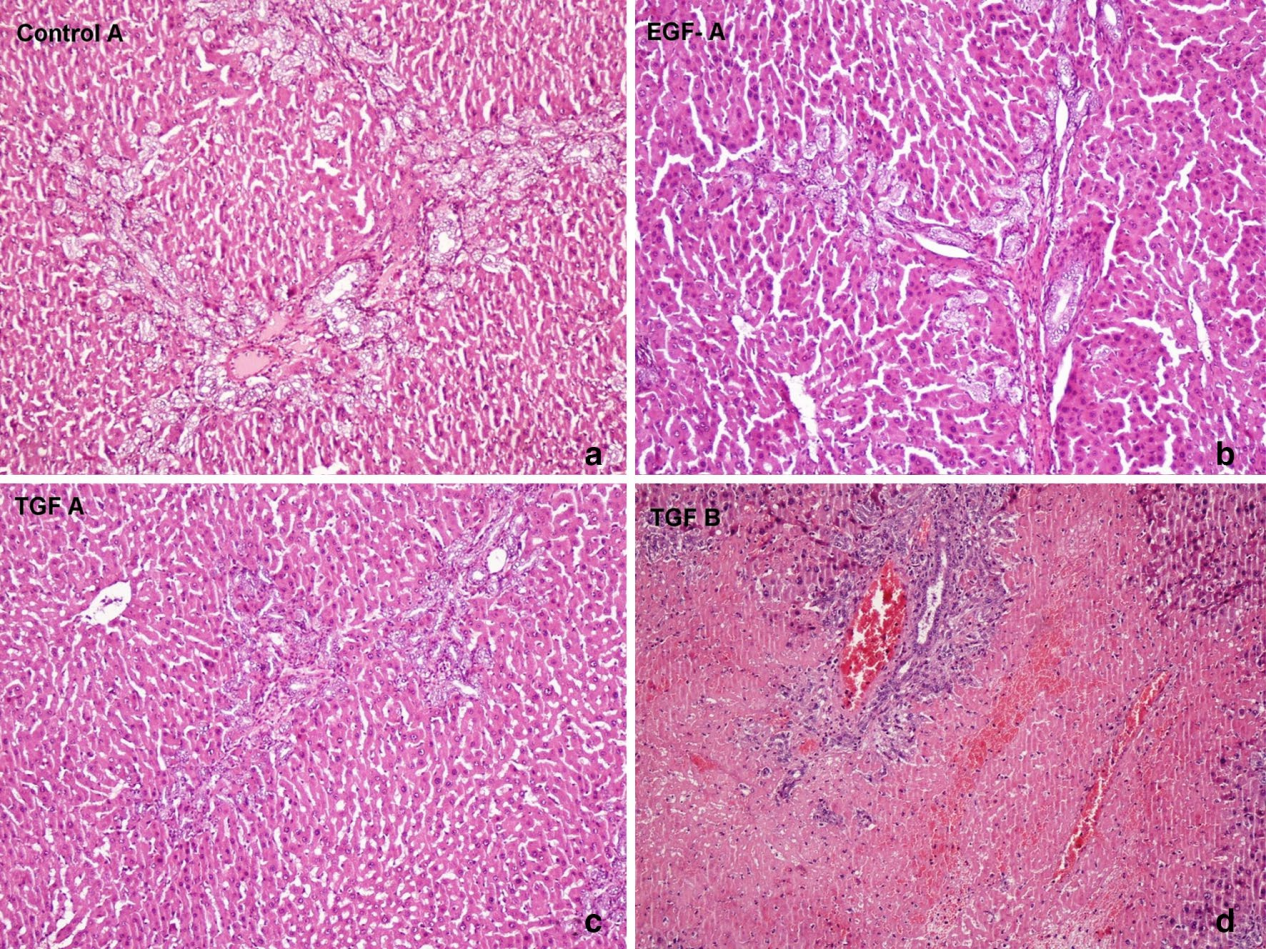
Histopathological evaluation of the liver tissues of rats (Hematoxylin and Eosin, ×100). **a** In control A group ductal proliferation score was significantly increased. Central necrosis, piecemeal necrosis and focal necrosis areas defined. **b** In EGF-A group. Fibrosis expansion areas seen in portal area. **c** In TGF-A group. Ductal proliferation and portal area fibsosis. **d** In TGF-B group. Ductal proliferation and portal area expansion

**Fig. 4:**
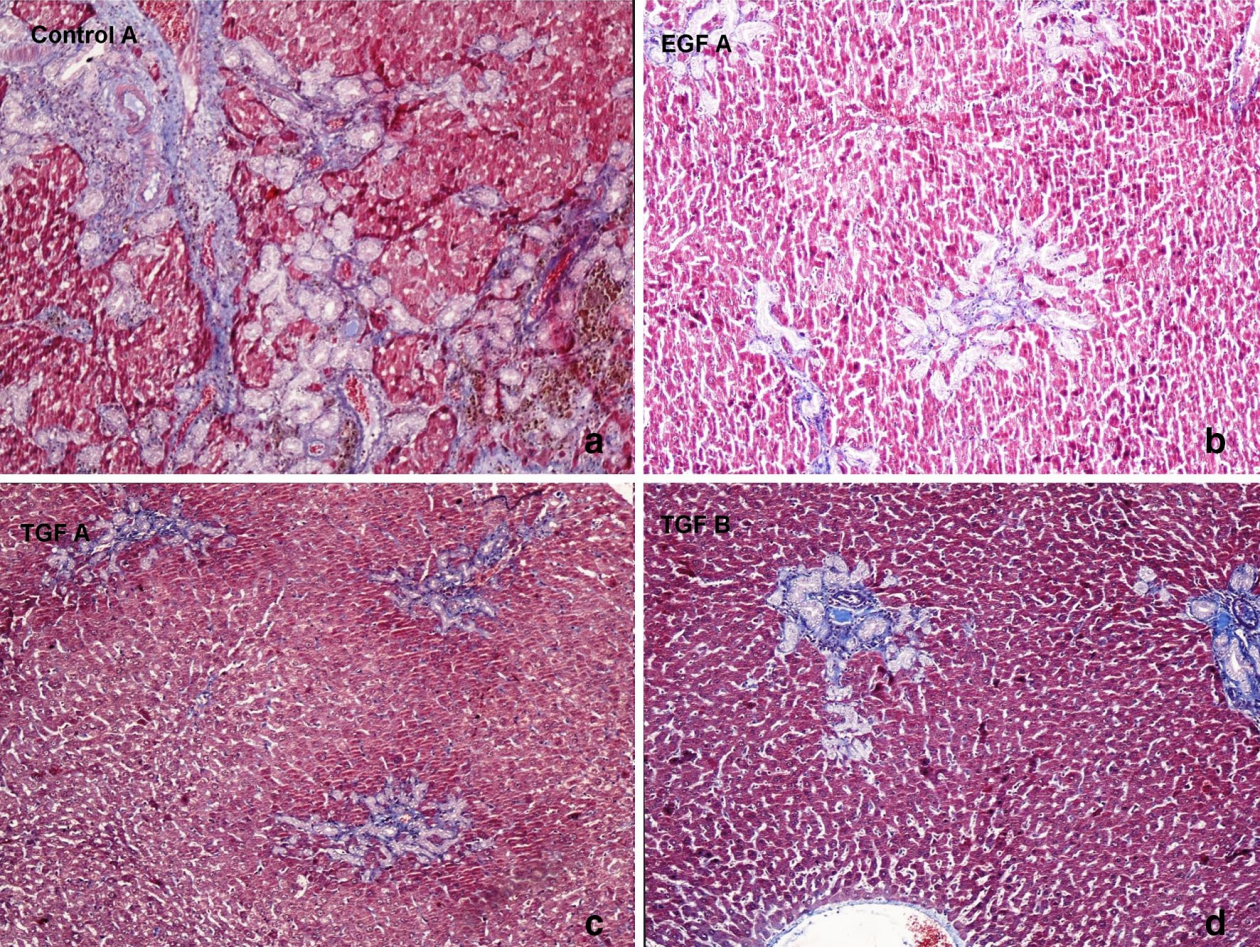
Histopathological evaluation of the liver tissues of rats (Masson’s Trichrome, ×100) **a** In control A group ductal proliferation score was significantly increased. Central necrosis, piecemeal necrosis and focal necrosis areas defined. **b** In EGF-A group. Fibrosis expansion areas seen in portal area. **c** In TGF-A group. Ductal proliferation and portal area fibsosis. **d** In TGF-B group. Ductal proliferation and portal area expansion

After H&E and Masson’s Trichrome staining, the liver tissues were evaluated and scored histological by the same pathologist, who was blinded to the experiment. The following sections were evaluated according to the modified histological activity index: portal inflammation, focal necrosis, confluent necrosis, piecemeal necrosis, apoptosis, and focal inflammation (Knodell et al. 1981; Ishak et al. 1995). Ductal proliferation was scored using the following grading system: 0 for <10 % of portal areas involved, 1 for 10–50 % of portal areas involved, 2 for >50 % of portal areas involved, 3 for a circumferential involvement of at least 50 % of the portal area without a significant expansion of the portal tract, 4 for a circumferential involvement of at least 50 % of the portal area with significant expansion of the portal tract 5 for the same criterion as level 4, plus a bridging of the portal tracts in <20 % of instances, and 6 for the same criterion as level 4 plus >20 % of the portal tracts showing bridging involvement (Table 3) (Sheen-Chen et al. 2006). Histopathological score results were listed in Table 2. In the Control A group, the ductal proliferation score was significantly increased. Central necrosis, piecemeal necrosis, and focal necrosis areas were defined and the histopathological findings of cholestasis were determined in both control groups, particularly in Control B group. Ductal proliferation scores were significantly decreased in the EGF A and TGF A groups, while the IGF A group showed a decreased ductal proliferation score. The ductal proliferation score of the IGF B group was significantly higher while that in the TGF B group was low. There was no significant difference between the scores of the reversible and irreversible jaundiced groups. In the HGF A group, peripheral fibrosis areas and ductal proliferation was observed. Similarly, in the EGF A group, fibrosis expansion areas were observed in the portal area. In the IGF A group, portal inflammation and piecemeal necrosis were determined, as well as ductal proliferation. Ductal proliferation and portal area expansion were seen in the TGF B group. The HGF A and HGF B groups showed similar histological findings. In addition to the EGF A group, focal necrosis and parancimal necrosis areas were observed in the EGF B group. There were no histological differences between the IGF A and IGF B groups. Ductal proliferation and portal area fibrosis in the TGF B group were less than in the TGF A group (Figs. 3, 4).

**Table 3:** Classification of scores based on the microscopic findings of liver tissue

Score	Findings
0	Proliferation in 10 % of portal areas
1	Proliferation in 10–50 % of portal area
2	Proliferation in more than 50 % of portal areas
3	At least 50 % of portal areas participated in an environmentally proliferation
4	With a significant expansion in the portal system; at least 50 % of portal areas participated in an environmentally proliferation
5	In addition to Score 4; bridging necrosis between portal areas; less than 20 %
6	In addition to Score 4; bridging necrosis between portal areas; more than 20 %

## Discussion

Cholestasis is a common clinical problem associated with bile duct obstructed diseases such as choledocholithiasis, periampullary cancer, and hepatocellular carcinomas. EGF and HGF have beneficial effects on liver injury and hepatic regeneration (Holmes et al. 2007; Noguchi et al. 1991; Berlanga et al. 1998). HGF, a potent mitogen for hepatocytes, is increased in various liver diseases. HGF has beneficial effects on hepatic cholestatic fibrosis (Li et al. 2007; Xia et al. 2006). Recent evidence has indicated that the growth hormone is involved in liver regeneration. In liver regeneration induced by partial hepatectomized mice, liver-specific IGF type 1 receptor knockout caused a significant decrease in hepatocyte proliferation in males (Desbois-Mouthon et al. 2006). TGF-beta 1 plays a role in the liver cell regeneration and atypical bile duct proliferation and in areas of rapidly progressing atypical bile duct proliferation (Takiya et al. 1995). Serum HGF levels decreased gradually after biliary drainage in patients who showed decreased serum bilirubin levels (Higaki et al. 1999).

Known growth factors play a role in liver injury and liver regeneration, as supported by experimental studies. However, the role of growth factors in posticteric liver damage and prolonged cholestasis is not certain. In clinical cases, determining whether a biliary drainage procedure before surgery is useful or not is also a matter of debate; there have not been enough studies on this subject (Makino et al. 2006). Patients are commonly referred to the hospital after jaundice develops. As a result, prolonged jaundice is not a rare clinical condition. In our study, we have tried to determine the role of the growth factors on liver damage in obstructive jaundiced rats. Well-known growth factors that affect liver generation and prophylactic effects on liver damage were included in our study: HGF, EGF, IGF-1, and TGF-ß1.

In the present study, we determined the effect of growth factors on liver damage during primary and established jaundice. Bile duct ligation continued for 5 days in irreversible jaundiced groups, as defined in the literature (Georgiev et al. 2008). Tests of liver function were studied as evidence of liver damage and to inform the degree of cholestasis. We observed that the liver function tests were higher in both the reversible and irreversible jaundiced control groups versus the sham group. Liver tissue MDA levels were significantly higher in the EGF A and IGF A groups (p < 0.05). Histopathological scores were lower in the EGF A and TGF A groups, but, however, higher in the IGF A group. It has been reported that the apoptotic index and the inflammatory response of liver fall away in EGF- and HGF-treated rats (Thatch et al. 2008). EGF has beneficial effects on cholestatic liver injury caused by bile duct ligation. EGF treatment was more effective in the irreversible group compared with the reversible group. IGF-1 was used in experimental obstructive jaundice to determine the apoptosis and ductular proliferation of liver tissue. After the administration of IGF-I, hepatocyte apoptosis was significantly diminished (Kirkland et al. 2010). However, in both the reversible and irreversible jaundiced groups; the IGF-1 treatment was ineffective for hepatic damage and posticteric liver injury. However, it is likely that there was no specific correlation between all reversible and irreversible jaundiced groups. In our study, histopathological results of IGF treatment groups were higher than in the control groups (p < 0.05). The Histopathological score of TGF B groups was lower than in the HGF B and Control B groups. Considering all the groups compared, there was no significant difference between the reversible and irreversible groups according to histopathological changes (p > 0.05).

As is known in the literature, hepatocyte apoptosis increased and parancimal and hepatocyte necrosis decreased in the liver tissue of common bile ligated rats treated with HGF (Li et al. 2007). According to all the parameters of our study, the HGF treatment was found to be effective in irreversible jaundiced rats, consistent with the literature. EGF and HGF modulated the hepatic inflammatory response and apoptotic index in this established liver-injury model and may diminish liver damage in patients with liver injuries (Thatch et al. 2008). EGF is responsible for liver regeneration (Yokoyama et al. 2007; Lai et al. 1992; Kiso et al. 1995; Rasmussen et al. 1992; Date et al. 2000). EGF treatment was effective in the irreversible jaundiced group versus the Control A group. The histopathological scores of the EGF A group was lower versus Control A group. After administration of IGF-1, the increased hepatocyte apoptosis and ductular proliferation after CBD ligation were significantly diminished (Sheen-Chen et al. 2006). The IGF-1 treatment was not effective in both treatment groups. Fibronectin production of both normal and injured hepatocyte was affected by the TGF-ß1 treatment (Kloek et al. 2008). TGF ß 1 treatment results in the regeneration of hepatocyte and increases the extracellular matrix in liver tissue. In histopathological studies, hepatocyte regeneration and ductal proliferation were detected; the scores of the TGF A and B groups were significantly lower than those of the control groups (p < 0.05). This finding is likely an early result of TGF Beta-1 treatment; later studies caused liver fibrosis. However, HGF treatment was effective in the HGF A group, but not in the HGF B group. Despite decreased levels of liver necrosis and biochemical assays, there was no significant difference between the HGF B group and the Control B group. A cholestatic rat is more susceptible to post ischemic liver injury and these injurious effects were significantly attenuated by biliary decompression. The antioxidant capacity of the liver was strongly decreased in cholestatic livers (Aldana et al. 1994). In the study of Aldana et al.; HGF mRNA was elevated following obstruction and showed increased expression 1 day after decompression, peaking at 2 days after repair. As a result, HGF may play a role in cellular proliferation during repair or may act as a compensatory growth factor during injury (Aldana et al. 1994).

## Conclusion

Growth factors have beneficial effects for preventing liver damage while obstructing jaundice. Furthermore, growth factors may play an effective role during the resolution of jaundice. We purpose that growth factors could protect the liver from cholestatic injury if the patient is diagnosed before sustained jaundice. In addition, liver damage will decrease after bile drainage. Before chronic cholestatic liver injury is fully realized, the maintenance of HGF and EGF levels prevents liver damage.

## Abbreviations

CBD: common bile duct
HGF: hepatocyte growth factor
EGF: epidermal growth factor
IGF: insulin-like growth factor
TGF: transforming growth factor
MDA: malondialdehyde
ALT: alanine aminotransferase
AST: aspartate aminotransferase
ALP: alkaline phosphatase
GGT: gamma-glutamyl transpeptidase
